# Radial Arteriovenous Fistula After a Left Heart Catheterization: Two Case Reports

**DOI:** 10.7759/cureus.38799

**Published:** 2023-05-09

**Authors:** Nkechi A Okam, Uzochukwu Ibe, Russell Stein, Ira Galin

**Affiliations:** 1 Internal Medicine, Danbury Hospital, Danbury, USA; 2 Cardiology, Danbury Hospital, Danbury, USA; 3 Cardiology, Danbury Hospital, Danbury , USA

**Keywords:** radial coronary angiography, radial arteriovenous fistula, sequelae of radial artery catheterization, arteriovenous fistula repair, distal radial artery access

## Abstract

The distal transradial artery (TRA) approach has been increasing in popularity over recent years due to its favorable ergonomics and potential for fewer vascular complications. Other advantages include lower bleeding risk, early ambulation, lower procedural costs, and same-day discharge, resulting in additional cost savings. We discuss two cases of patients who underwent left heart catheterizations through the radial artery access site and afterwards experienced fistula formation. Our case series brings to light a rare occurrence of arteriovenous fistulas (AVFs) following cardiac catheterization via the transradial artery site, thus enhancing our knowledge of the risk associated with this access site. The pathophysiology of AV fistula remains the same regardless of transfemoral or transradial artery use. During the procedure, needle diversion into the venous tributary results in an unrecognized combined artery and vein puncture, which usually seals spontaneously. However, if the communication persists, an AV fistula may occur. The majority of patients who suffer from an iatrogenic AVF as a result of TRA do not develop clinical signs of hemodynamic significance. There are various therapeutic strategies, which include surgical repair, placement of a covered stent, ultrasound-guided compression of the AV fistula, and conservative management. Both of our patients were evaluated by vascular surgery; one of the patients found the constant pulsation and bruit burdensome and underwent surgical repair.

## Introduction

The distal transradial artery (TRA) approach has been increasing in popularity over recent years due to its favorable ergonomics and potential for fewer vascular complications [1]. Other advantages include lower bleeding risk, early ambulation, lower procedural cost, and same-day discharge, resulting in additional cost savings [2]. Our case series reveals an uncommon occurrence of arteriovenous (AV) fistulas after cardiac catheterization via the transradial artery site, thereby advancing our understanding of the risk connected with this access site. An AV fistula is an abnormal connection between an artery and a vein. Iatrogenic AV fistulas are a rare complication of TRA, which can be attributed to the fact that only small veins are present in close proximity to the radial artery [2]. This article was previously presented as a meeting abstract at the 2023 ACC/WCC American College of Cardiology/World Congress of Cardiology Meeting on March 5, 2023.

## Case presentation

Case 1

**Figure 1:**
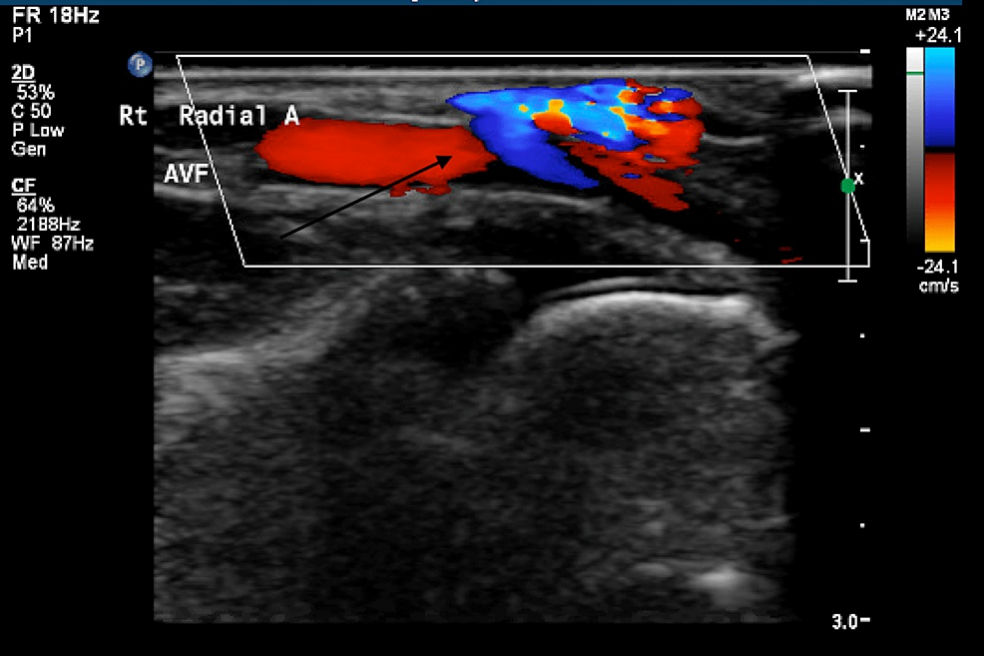
AV fistula between radial artery and a vein.

A 51-year-old female with a past medical history of a prior MI presented with mild substernal chest pain similar to a prior myocardial infarction. Six months prior, she had undergone coronary stenting with two drug-eluting stents in her right coronary artery. The procedure was done via the right radial artery approach; heparin was chosen for anticoagulation to avoid clot formation during the procedure; and hemostasis was achieved using a 24 cm Vasc Band Hemostat Regular device with no immediate complications. However, over the following months, she occasionally experienced paresthesia in the fingers of her right hand. She also noted that she could hear her pulse when she placed her head on her right arm to sleep. On presentation, she had a blood pressure of 140/90 mmHg and a heart rate of 69 bpm. Her EKG showed a normal sinus rhythm without acute ST-T wave abnormalities, and her fifth-generation troponin was normal. She had normal heart sounds, a regular rhythm, and no murmurs on the exam. She had a notable, palpable thrill and an audible right radial bruit. An ultrasound was performed and showed a radial arteriovenous fistula (Figure 1). She was referred to vascular surgery.

Case 2

**Figure 2:**
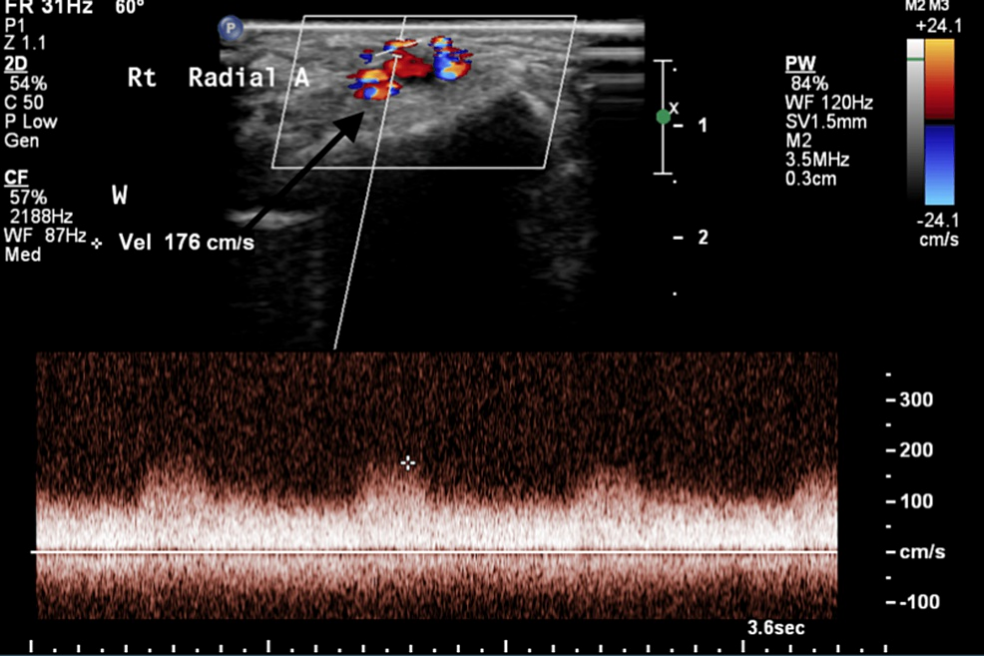
Radial artery demonstrating spectral broadening and diastolic flow seen in arterial beds with low resistance outflow.

A 72-year-old female with a history of hypertension, hyperlipidemia, paroxysmal supraventricular tachycardia (SVT) with an ablation, stress-induced cardiomyopathy, and non-obstructive computer-aided design (CAD) presented for a routine office visit. She had undergone a cardiac catheterization via radial artery approach two years prior. During the procedure, heparin was used for anticoagulation, and hemostasis was achieved using a 29-cm-long Vasc Band Hemostat device. During the office visit, she reported difficulty sleeping on her right side because the pulse in her arm was significantly greater than on the left side, and she was able to hear her pulse. She had no other associated complaints of pain, numbness, or limitations with the use of the hand. She had an ultrasound of her upper extremity that showed a radial-cephalic fistula at the right wrist (Figure 2). She was also seen for vascular surgery and ultimately underwent ligation of the AV fistula.

## Discussion

The pathophysiology of AV fistula remains the same independent of transfemoral or transradial artery use. During the procedure, needle diversion into the venous tributary results in an unrecognized combined artery and vein puncture, which usually seals spontaneously. However, if the communication persists, an AV fistula may occur [3]. Some local indicators for puncture site complications may include hematoma, arterial spasm, excessive bleeding, or swelling. Despite the rarity of AV fistulas following transradial access sites, the growing popularity of coronary interventions may contribute to an increase in their occurrence, emphasizing the importance of proper diagnosis and early intervention [4]. Other potential procedural complications include radial artery occlusion, non-occlusive radial artery injury, radial artery pseudoaneurysm, and radial artery spasm [2]. Persistent AV fistulas, especially if large, can predispose to a hemodynamic shift from a higher systemic vascular resistance in the artery to a lower resistance in the venous system, potentially leading to high-output cardiac failure; limb ischemia may also be seen.

The RIVAL trial is the largest randomized trial comparing radial versus femoral access for coronary angiography and intervention [5]. It included 7,021 patients with acute coronary syndrome (ACS) undergoing either of the two procedures. Although there was no statistically significant difference in the primary outcome of death, myocardial infarction, stroke, or non-coronary artery bypass graft-related bleeding at 30 days between the radial and femoral approaches, major vascular complications such as large hematomas, pseudoaneurysms, AV fistulas, and ischemic limbs requiring surgery occurred in 1.4% of radial access compared to 3.8% of femoral access (P<0.001). An investigator further observed iatrogenic AVF in 5 (0.14%) of 3,514 patients in the transfemoral group and none of 3,507 patients in the transradial group [5]. Kelm et al. found significant predictors of AV fistula to be high heparin dosage, Coumadin therapy, puncture of the left groin, arterial hypertension, and female gender [6].

The majority of patients who suffer from an iatrogenic AVF as a result of TRA do not develop clinical signs of hemodynamic significance [1]. There are various therapeutic strategies, which include surgical repair, placement of a covered stent, ultrasound-guided compression of the AV fistula, and conservative management [3].

## Conclusions

Both of our patients underwent left heart cardiac catheterizations via the right distal radial artery access site, and AV fistulas developed likely as a result of simultaneous venous puncture during arterial access, because of the close anatomic relationship between the cephalic vein and the radial artery as it passes through the anatomic snuffbox. Both our patients were evaluated by vascular surgery and one of them found the constant pulsation and bruit burdensome; and underwent surgical repair.
